# Small-Scale Preparation of Fluorescently Labeled Chemical Probes from Marine Cyclic Peptides, Kapakahines A and F

**DOI:** 10.3390/md19020076

**Published:** 2021-01-31

**Authors:** Rie Kamihira, Yoichi Nakao

**Affiliations:** 1 Research Institute for Science and Engineering, Waseda University, 3-4-1 Okubo, Shinjuku-ku, Tokyo 169-8555, Japan; r-k-cmk@ruri.waseda.jp; 2 School of Advanced Science and Engineering, Waseda University, Shinjuku-ku, Tokyo 169-8555, Japan

**Keywords:** marine natural products, cyclic peptides, fluorescent probe, chemical biology

## Abstract

A number of bioactive marine natural products have been isolated so far, but it is still difficult to disclose their modes of action. In this study, we prepared fluorescently labeled chemical probes from the cytotoxic marine cyclic peptides kapakahines A (**1**) and F (**2**) to visualize their localization as the first step of the study of their modes of action. We used fluorescent dyes **3a** or **3a/b** (a 1:1 mixture of **3a** and **3b**) whose terminal *N*-hydroxysuccinimide (NHS) group can react with the free amino groups of kapakahines. The fluorescently labeled kapakahine A (Kap A-5-FL, **5a**) stained P388 murine leukemia cells and HeLa human cervical cancer cells, while cells treated with fluorescently labeled kapakahine F (Kap F-5-FL, **6a**) only weakly stained them. Further analysis of the confocal images of the stained cells with higher magnification (×100) indicated the localization of Kap A-5-FL (**5a**) in the cells. In this paper, we report the small-scale preparation and a new delivery method of fluorescent probes, as well as the application of these procedures to cell staining.

## 1. Introduction

Marine organisms are known as a rich source of bioactive secondary metabolites and among these, cyclic peptides constitute one of the major categories of unique structures and bioactivities [1,2,3,4,5]. However, it still remains difficult to elucidate the modes of action for marine cyclic peptides. Fluorescently labeled probes are powerful tools to determine the localization of natural products in cells. Affinity bead-linked chemical probes are essential for the identification of target molecules. In the preparation of such useful chemical probes, scarce availability of marine natural products often becomes the obstacle. To overcome this problem, total synthesis of chemical probes based on the natural products is the best solution. However, not all the natural products are suitable for total synthesis and it usually takes a long time. Therefore, it is desirable to apply a simple and efficient small-scale preparation procedure for the scarce amounts of “natural” compounds as well [6,7,8,9].

**Figure 1:**
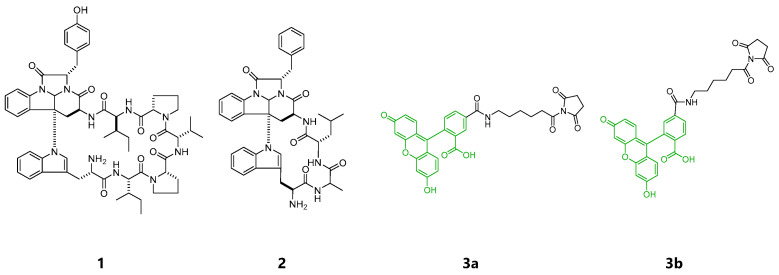
Structures of kapakahines A (**1**), F (**2**), 5-SFX (**3a**) and 6-SFX(**3b**).

Aurilide, a cytotoxic cyclic depsipeptide isolated from the sea hare *Dolabella auricularia*, is an excellent example of the successful identification of a target protein and the localization in cells using chemical probes provided by total synthesis [10,11]. Cytotoxic and actin-binding marine natural products, aplyrorine A [12,13,14] and kabiramide C [15,16] were fluorescently labeled and successfully visualized the localization of the probes in cells. To add more examples that successfully disclose modes of action for marine cyclic peptides, we turned to kapakahines A (**1**) and F (**2**) isolated from a marine sponge *Cribrochalina olemda* [17,18] (Figure 1). Both compounds contain a common fused α-carboline core composed of Trp and Phe/Tyr residues. Kapakahine A showed cytotoxicity against P388 murine leukemia cells with an IC_50_ value of 5.4 μg/mL, while kapakahine F showed only weak cytotoxicity at 5.0 μg/mL [17,18,19]. In spite of their unique structures, their detailed biological functions or modes of action are still unknown. In 2015, Rocha et al. prepared fluorescent probe of kapakahine E, an analogue of **1**, by derivatization at the free amino group of the synthetically prepared kapakahine E with the fluorescent coumarin, and visualized its localization in Golgi apparatus in cells [20]. Although total synthesis of smaller sized kapakahines B, E, and F has been achieved [21,22,23,24,25], the most potent and largest kapakahine A has not yet been synthesized. As the result, the fluorescent probe of kapakahine A (**1**), which also contains a free amino group, has not been prepared yet. 

In this paper, we report the small-scale preparation of fluorescent probes of “natural” kapakahine A and “synthetic” kapakahine F (because of the unavailability from the remaining “natural” kapakahine F), as well as the development of a delivery procedure for these fluorescent probes with low solubility. Application of this delivery procedure to the fluorescent probes of kapakahines successfully visualized the localization sites of these probes. 

## 2. Results

### 2.1. Preparation of Fluorescent Probes of Kapakahines 

At first, fluorescent probes of kapakahines A (Kap A-5,6-FL; **5a/b**) and F (Kap F-5,6-FL; **6a/b**) were prepared under the conditions shown in Scheme S1. For the fluorescent labeling, we used a commercially available 1:1 mixture of 6-(fluorescein-5 or 6-carboxamido) hexanoic acid succinimidyl esters (5- or 6-SFX: **3a/b**) as the fluorescent dyes whose *N*-hydroxysuccinimide (NHS) groups can react with the amino groups of kapakahines under simple and mild conditions [26]. Reversed-phase HPLC (high-performance liquid chromatography) purification of the reaction mixture derived from the conjugation of **3a/b** with kapakahines A (**1**, 1.0 mg) or F (**2**, 0.5 mg) provided Kap A-5,6-FL (**5a/b**, 340 µg) and Kap F-5,6-FL (**6a/b**, 79 µg) as inseparable mixtures, respectively. Fluorescent dye **3a/b** (2.9 mg) was also treated with MeOH giving methyl esters **4a** (0.8 mg) and **4b** (0.7 mg) that were separable in the reversed-phase HPLC. 

**Scheme 1:**
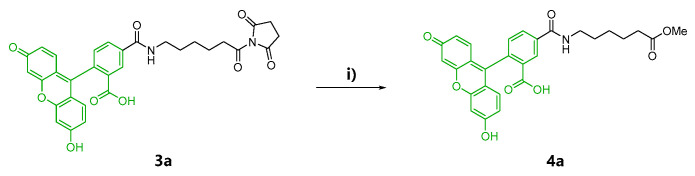

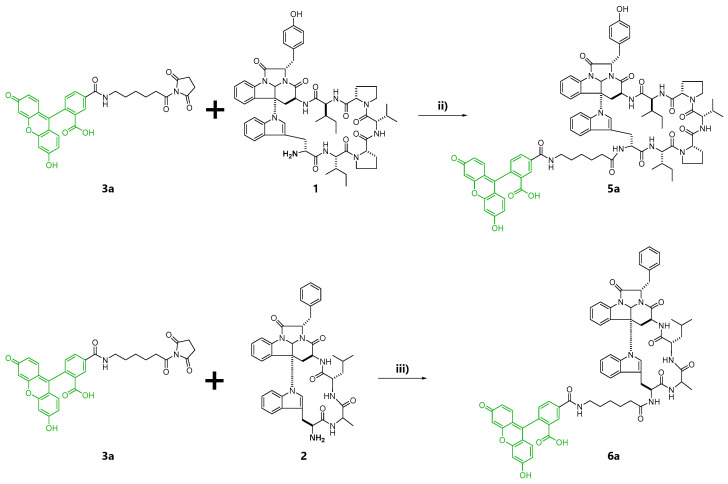
Reaction conditions for control **4a**, as well as chemical probes **5a** and **6a**. (**i**) MeOH, triethylamine, rt, 20 h; (**ii**) DMF (*N,N*-dimethylformamide), rt, 20 h; (**iii**) DMF, rt, 20 h.

In the second preparation, probes solely containing a fluorescein-5-carboxamido group, Kap A-5-FL (**5a**) and Kap F-5-FL (**6a**), were prepared. Compound **4a** was also prepared as the negative control in the cell staining experiments (Scheme 1).

### 2.2. Cell Staining

Localization of kapakahines was first investigated by cell staining using the mixed fluorescent probes (**5a/b** and **6a/b**). Two types of cancer cells (HeLa human cervical cancer cells and P388 mouse leukemia cells) were chosen for cell staining. Kapakahine A (**1**) was reported to show cytotoxicity against P388 cells with an IC_50_ value of 5.4 μg/mL (equivalent to 5.1 μM, not reported for HeLa cells), while kapakahine F (**2**) showed only weak cytotoxicity at 5.0 μg/mL against both cells. Based on the cytotoxicity, the final concentration of Kap A-5,6-FL (**5a/b**) and Kap F-5,6-FL (**6a/b**) were set to 20 μM so as to maintain enough affinity to the target molecules in cells. HeLa cells were seeded into each well of a 96-well culture plate. After incubation for 20.5 h, 100 µL of the medium containing the probe was administered into each well and cultured for additional 20 h. The results showed that Kap A-5,6-FL (**5a/b**) stained HeLa cells, while only weak fluorescent staining was observed for Kap F-5,6-FL (**6a/b**). However, microscopic analysis gave low contrast images because the bottoms of the wells were also fluorescently stained with precipitated **5a/b** or **6a/b** (Figure S6).

**Figure 2:**
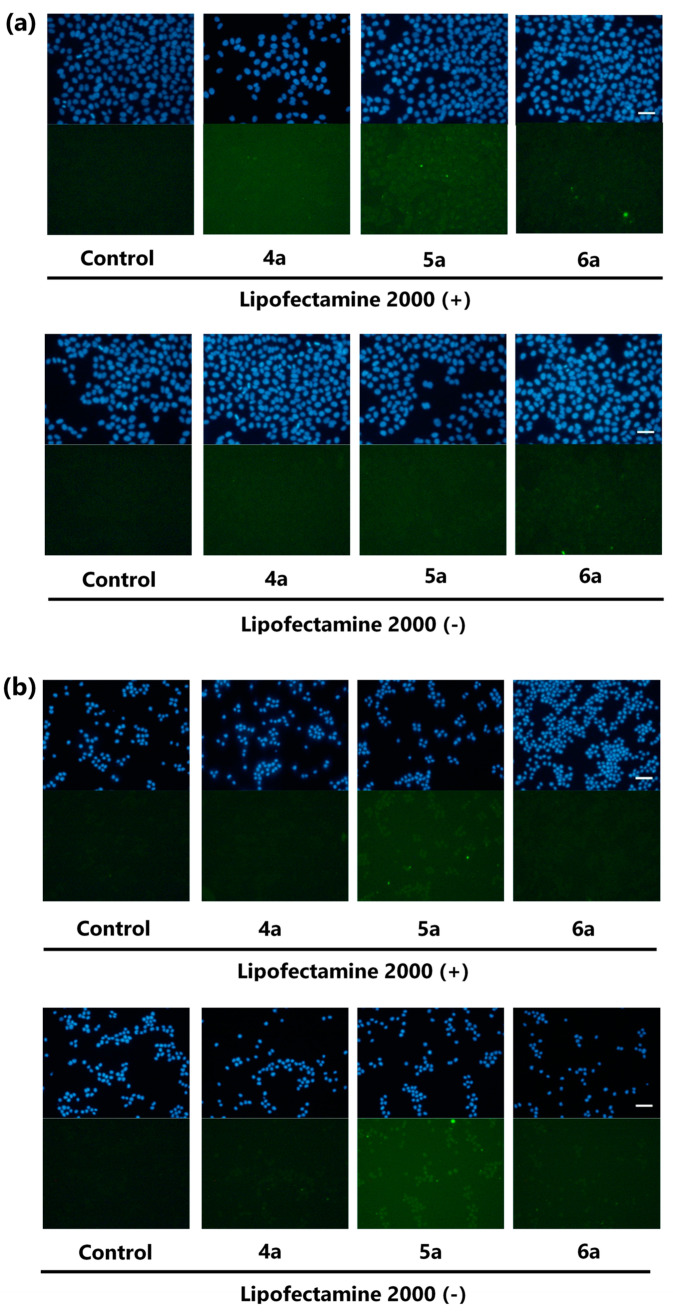
Microscopic images of HeLa cells (**a**) or P388 cells (**b**) treated with EtOH (control), **4a** (negative control), **5a**, and **6a** with or without Lipofectamine 2000 for 2 h (green). Nuclei stained with Hoechst 33,342 (blue), magnification ×20, scale bar: 50 μm. The brightness and contrast were adjusted equally for all the green fluorescent images.

To reduce the undesired staining caused by the poor solubility of the probes, we attempted to prepare Kap A-5-FL (**5a**) and Kap F-5-FL (**6a**) reacted with 5-substituted fluorescent dye 5-SFX (**3a**) solely. However, to our great disappointment, there was more precipitation of the crystalline probes **5a** and **6a** covering the bottom of the wells (Figure S7). Removal of the precipitation from the probe solution by standing for 45 min before addition reduced the amount of the crystals and the background staining. However, cells were not stained either, suggesting there were not enough quantity of probes dissolved in the supernatant of the solution. Dramatic improvement in the solubility and the cell permeability was achieved by treatment of **5a** and **6a** with Lipofectamine 2000, a transfection reagent (Figure S8). With this “Lipofectamine system”, Kap A-5-FL (**5a**) stained HeLa cells more clearly, with less precipitation. Microscopic images of HeLa and P388 cells treated with **5a** and **6a** revealed that **5a** stained both cells but **6a** faintly stained only HeLa cells (Figure 2).

**Figure 3:**
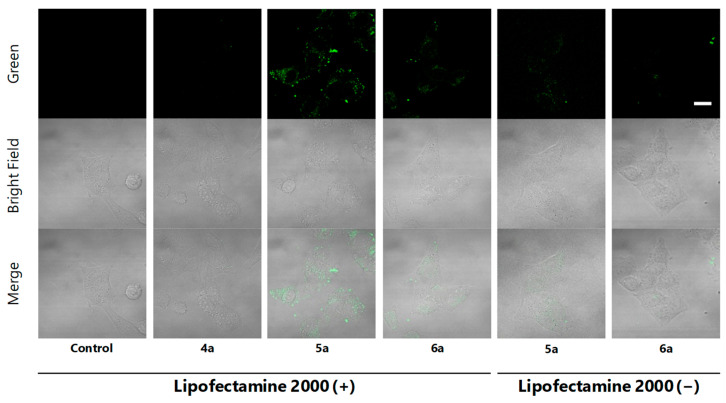
Confocal microscopic images of HeLa cells incubated with EtOH, **4a**, **5a** and **6a** with or without Lipofectamine 2000 (green) for 2 h (green, upper lane), bright field (middle lane), and the merge (lower lane), magnification ×100, scale bar: 20 μm. The brightness and contrast were adjusted equally for all the green fluorescent images.

To confirm cell staining by **5a** and **6a** and to obtain images with higher resolution, HeLa cells treated with EtOH (control), methyl ester **4a** (negative control) and fluorescent probes **5a** and **6a** using “Lipofectamine system” were analyzed under the confocal microscope. As a result, localization in the cytoplasm granules in cells was clearly indicated for Kap A-5-FL (**5a**), while cells treated with **6a** gave weaker and different localization images (Figure 3 Lipofectamine 2000 (+)). This observation is consistent with the superior cytotoxicity of kapakahines A (**1**) over F (**2**), suggesting their appropriate localization in cells. Microscopic images treated with **5a** and **6a** without “Lipofectamine system” did not show clear localization images, suggesting the efficacy of this system (Figure 3; Lipofectamine 2000 (−)). 

**Figure 4:**
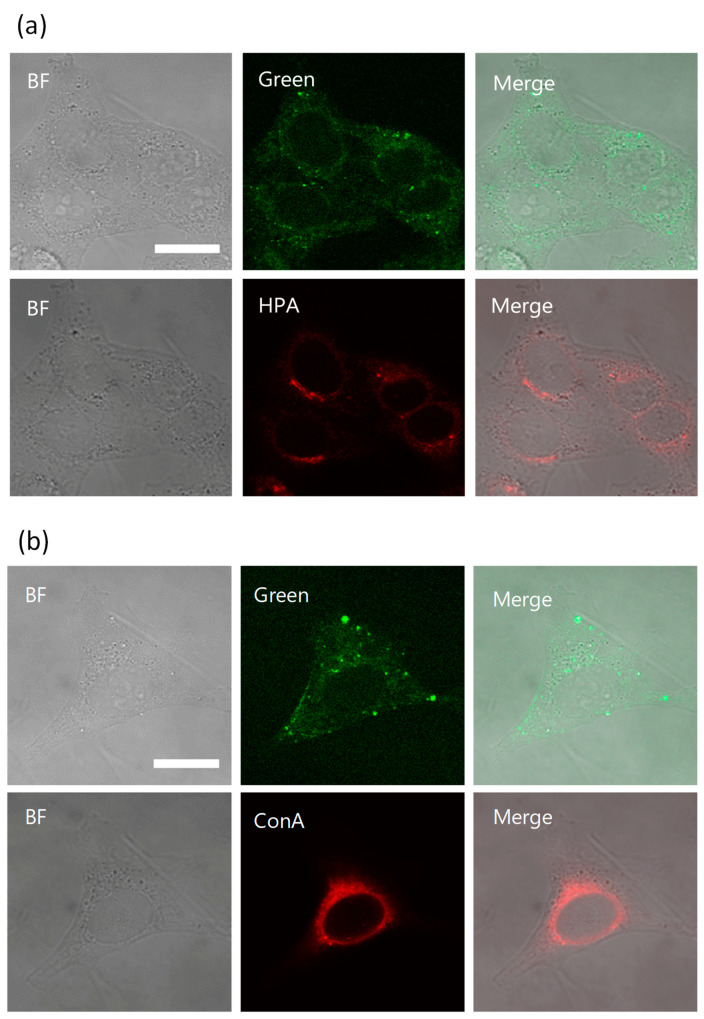
Co-localization study in HeLa cells. Confocal microscopic images of HeLa cells incubated with **5a** in Lipofectamine 2000 for 2 h (green). After incubation with **5a** (upper lane) for 2 h and the same cell stained with Lectin HPA (lower lane) (**a**). After incubation with **5a** (upper lane) for 2 h and the same cell stained with concanavalin A (ConA) (lower lane) (**b**). Bright field (BF, left) and HPA (red, middle) or ConA (red, middle), and merged images (right). Golgi stained with HPA Alexa fluor 647 conjugate (red), endoplasmic reticulum stained with ConA Alexa fluor 594 conjugates (red) respectively, magnification ×100, scale bar: 20 μm. The brightness and contrast were equally adjusted for all the green fluorescent images. **5a** was added to living cells for 2 h, fixed with 4% PFA, and fluorescent images were obtained (upper lane). Staining with Con A and HPA was performed after permeabilization of the fixed cells (lower lane).

Rocha et al. revealed that kapakahine E fluorescent probe (Kap E-FL) was localized in the Golgi apparatus. Based on this observation, localization images in HeLa cells of KapA-5-FL (**5a**) and Golgi-binding lectin, HPA [*Helix pomatia* (edible snail) agglutinin] Alexa Fluor 647 conjugate (Thermo Fisher Scientific Inc., Waltham, MA, USA) [20] were compared. In this experiment, the treatment of the permeabilization buffer, containing Triton X-100 damaged the fluorescent staining by **5a**. Therefore, the fluorescent images treated with **5a** were first obtained after the fixation, and then cells were permeabilized and stained by HPA Alexa Fluor 647 conjugate. The same cells whose microscopic images were captured for **5a** were manually searched under the microscope, then fluorescent images of the same cells with HPA staining were obtained. Comparison of the images treated with Kap A-5-FL (**5a**) and HPA Alexa fluor 647 conjugate in cells did not show their co-localization in Golgi apparatus (Figure 4a, Figure S10). This observation is different from that in the case of Kap E-FL, and may suggest that kapakahine A (**1**) did not bind to the same target molecule of Kapakahine E-FL. However, we could not confirm this hypothesis because there could be effects from the different fluorescent dye (7-dimethylamino-4-coumarin acetamide instead of fluorescein) introduced to kapakahine E. 

Concanavalin A (ConA) Alexa Fluor 594 conjugate (Thermo Fisher Scientific Inc., Waltham, MA, USA) was used for staining of endoplasmic reticulum and again the obtained images for the stained cells with **5a** did not co-localize in endoplasmic reticulum (Figure 4b, Figure S10).

## 3. Discussion 

In this study, we prepared fluorescent probes Kap A-5,6-FL (**5a/b**) and Kap F-5,6-FL (**6a/b**) as well as Kap A-5-FL (**5a**) and Kap F-5-FL (**6a**) from marine cyclic peptides kapakahines A (**1**) and F (**2**), respectively, by introducing a fluorescent pigments **3a/b** or **3a**. 

This preparation applied a simple and efficient method suitable for labeling natural products on a small scale, but there were still some difficulties. For the preparation, the first difficulty was the method of weighing the μg order sample amounts after HPLC separation of the reaction products. We overcame this issue and obtained the accurate weights following the procedure described in Supplementary Materials. This is a simple but reliable method to measure the accurate concentration of the solution. 

The second difficulty was the poor solubility of the fluorescent probes in the cultural medium. The pure form of Kap A-5-FL (**5a**) prepared from a single fluorescent dye **3a** showed unexpectedly poorer solubility than that from a mixture of **5a/b**. Presumably, pure **5a** can aggregate easier and form crystals. Although pure **5a** showed lower solubility in the culture medium than the mixture **5a/b**, treatment of **5a** with Lipofectamine 2000 showed improvement in solubility. It is speculated that **5a** became more hydrophilic because of the encapsulating effects by Lipofectamine 2000. The highly hydrophobic **5a** and **6a** form a complex with the cationic lipid Lipofectamine, allowing better dissolution in the medium. The hydrophobicity of the natural compounds can be affected largely by fluorescent labelling, but this procedure recovers the reduced solubility and improves cell staining efficiently. 

We performed staining of HeLa cells as well as P388 cells using prepared Kap A-5-FL (**5a**) and Kap F-5-FL (**6a**) treated with Lipofectamine 2000. At the concentration of 20 μM, **5a** stained HeLa cells within 1 and 2 h, while **6a** stained HeLa cells only weakly. To obtain higher resolution images of the localization, confocal microscopic images of the HeLa cells treated by **5a** were analyzed. The obtained images showed that **5a** clearly localized in the cytoplasm, but not in the Golgi apparatus, on the basis of co-staining with HPA (Figure 4). This observation is different from the result by Rocha et al. in which Kap E-FL localized in Golgi apparatus [16], suggesting kapakahine A targets the biomolecule different from that of kapakahine E. 

To approach the modes of action, labelling of the bioactive compound is an important step. Labelling by RI [27], photoaffinity reaction [28], click reaction [29] and fluorescent dyes are major labelling methods. Although there are many choices in labelling methods, we often face difficulty to choose suitable labelling methods applicable to the limited quantity of the compounds in the case of marine natural products. Preparation of chemical probes using synthetically prepared natural products is the desirable way, but it requires multiple steps of reactions, e.g., Yakua'amide B [30]. Compared to the approach using synthetically prepared natural compounds available over 100 mg [31,32], the reaction scale dependent on the naturally available compounds has to be much smaller (below 1 mg). Our method may be useful for the preparation of probes using marine natural products with amino groups that have not yet been synthesized due to their structural complexity.

In this study, we prepared fluorescent probes using a naturally available small amount of kapakahines A (1, 0.5 mg) as well as the equivalent small amount of the synthetic F (**2**, 0.5 mg). Kap A-5-FL (**5a**) successfully visualized the in-cell localization. It is noteworthy that this method can be applicable to the preparation of the chemical probes for target identification that are linked to beads with active ester modification on the surface. 

## 4. Materials and Methods 

### 4.1. General Experimental Procedures

All nuclear magnetic resonance (NMR) spectra were recorded on Bruker Avance (600 MHz, Bruker Corporation, Billerica, MA, USA) spectrometers. Fluorescent microscopic images were obtained using an Olympus IX71 microscope equipped with an Olympus DP72 digital camera (Olympus Corporation, Tokyo, Japan). Confocal microscopic images were obtained using an Olympus FV1000 (Olympus Corporation, Tokyo, Japan). HPLC (high-performance liquid chromatography) was conducted using a JASCO PU-1580 pump system equipped with a JASCO FP-2020 Plus fluorescence detector (JASCO Corporation, Tokyo, Japan) or TOSOH UV8011 (TOSOH Corporation, Tokyo, Japan) or JASCO UV-4075 UV detectors (JASCO Corporation, Tokyo, Japan). Small amounts of probes were weighed using Sartorius SE2 Ultra Micro Balance (Sartorius, Göttingen, Germany). All the high-resolution mass spectra (HRMS) were acquired on a JEOL JMS-T100CS spectrometer (JEOL Ltd., Tokyo, Japan) at the Materials Characterization Central Laboratory, Waseda University, Tokyo, Japan.

### 4.2. Methods

#### 4.2.1. Synthesis of 6-(Fluorescein-5-Carboxamido) Hexanoic Acid, Methyl Ester (5-FL-COOMe: **4a**)

6-(Fluorescein-5-carboxamido) hexanoic acid succinimidyl ester (5-SFX: **3a**, 1.3 mg, 2.2 µmol, Thermo Fisher Scientific Inc., Waltham, MA, USA) were mixed with 1 µL of triethylamine (TEA, 7.2 µmol) in 645.6 µL of MeOH. The reaction mixture was stirred at room temperature in the dark for 20 h. The reaction was quenched with excess MeOH and the dried reaction mixture was subjected to reversed-phase HPLC [COSMOSIL 5C_18_-ARⅡ (φ10 × 250 mm, Nacalai Tesque Inc., Kyoto, Japan), flow rate; 2 mL/min, isocratic elution with 35% acetonitrile 0.05% TFA, detection; 220 nm, JASCO UV4075] yielding 5-FL-COOMe (**4a**, 550 µg). Compound **4a** gave an [M − H]^−^ ion peak at *m/z* 502.1511 (calcd. C_28_H_24_NO_8_ 502.1507) in the ESIMS.

Methyl esters **4a** and **4b** were also obtained by HPLC separation of the reaction mixture obtained following the same procedure but with a 1:1 mixture of 5- and 6-SFX (**3a/b**) instead of **3a** with adjustment of the amounts of triethylamine.

#### 4.2.2. Preparation of Kap A-5-FL (**5a**)

A portion (100 μL) of **3a** in *N,N*-dimethylformamide (DMF) (20 mg/mL, 6.8 μmol) was added dropwise to kapakahine A (**1**, 0.5mg, 0.5 μmol) dissolved in 50 μL of DMF. In this experiment, we used kapakahine A isolated from natural sources. The reaction mixture was stirred at room temperature in the dark for 20 h. After quenching with MeOH, the reaction mixture was subjected to ODS flash column chromatography eluting with 100% MeOH. The 100% MeOH eluting fraction was further purified by reversed-phase HPLC [COSMOSIL 5C_18_-ARⅡ (φ10 × 250 mm), flow rate; 2 mL/min, isocratic elution with 55% acetonitrile 0.05% TFA, detection; 220 nm, JASCO UV4075] to yield Kap A-5-FL (**5a**: 340 µg). In the ESIMS analysis, **5a** gave an [M − H]^−^ ion peak at *m/z* 1522.6737 (calcd. C_85_H_92_N_11_O_16_ 1522.6729).

Kap A-5,6-FL (**5a/b**) was also prepared following the same procedure but with **3a/b** instead of **3a.**

#### 4.2.3. Preparation of Kap F-5-FL (**6a**)

Kapakahine F (**2**, 0.5 mg, 0.7 μmol) in 33.3 μL DMF was added to 66.6 μL of the solution of reagent **3a** in DMF (20 mg/mL) and was stirred at room temperature in the dark for 20 h. In this experiment, we used synthetic kapakahine F (provided by Prof. Phil Baran). After quenching with MeOH, the fraction was further purified by reversed-phase HPLC [COSMOSIL 5C_18_-AR II (φ10 × 250 mm), flow rate; 2 mL/min, isocratic elution with 50% acetonitrile 0.05% TFA, detection; 220 nm JASCO UV4075] yielding Kap F-5-FL (**6a**, 370 µg) which gave an [M − H]^−^ ion peak at *m/z* 1171.4579 (calcd. C_67_H_63_N_8_O_12_ 1171.4571).

Kap F-5,6-FL(**6a/b**) was also obtained following the same procedure but with **3a/b** instead of **3a.**

#### 4.2.4. Cell Culture

Dulbecco’s Modified Eagle Medium (DMEM, Low Glucose, FUJIFILM Wako Pure Chemical Corporation, Osaka, Japan) supplemented with 10% fatal bovine serum (FBS, Biowest, Nuaillé, France), 10 µg/mL of antibiotic antimycotic and 2 µg/mL of gentamicin solution was used as the culture media for HeLa human cervical cancer cells. Roswell Park Memorial Institute medium (RPMI, FUJIFILM Wako Pure Chemical Corporation, Osaka, Japan) supplemented with kanamycin sulfate and HRDS solution (2,2’-dithiobisethanol) was used as the culture media for P388 murine leukemia cells. Both cells were cultured at 37 °C under the atmosphere of 5% CO_2_.

#### 4.2.5. Preparation of the Sample Solution for Cell Staining Using Lipofectamine 2000

Compounds **4a**, **5a**, and **6a** were dissolved in EtOH at the concentration of 2 mM, respectively. EtOH was used as the blank. Each portion (20 µL) of the solutions was mixed with 20 µL of 1 mg/mL of Lipofectamine 2000 (Thermo Fisher Scientific Inc., Waltham, MA, USA) in 1.5 mL microtube and then incubated for 10 min. Each solution (4 µL) was added to the cultural medium (200 µL) in each well of the 96-well glass bottom microplate (final concentrations of the **4a**, **5a** and **6a** were 20 µM in the medium), respectively. After pipetting, the plate was left to stand for 45 min at the room temperature. After 45 min, the supernatant was collected and used as the sample solution. The same preparation was performed for the sample without Lipofectamine 2000.

#### 4.2.6. Cell Staining

HeLa cells (10,000 cells/well in 200 µL medium) were plated into each well of a 96-well glass bottom microplate. After 24 h incubation, the medium in each well was removed and exchanged with 100 µL of sample solution prepared as above, then incubated at 37 °C under the atmosphere of 5% CO_2_ for 2 h. After incubation, the cells were fixed in 4% paraformaldehyde (FUJIFILM Wako Pure Chemical Corporation, Osaka, Japan) at room temperature for 20 min. After washing with phosphate-buffered saline (PBS, FUJIFILM Wako Pure Chemical Corporation, Osaka, Japan), nuclei were stained with Hoechst 33342 (1:1000, DOJINDO, Kumamoto, Japan) dissolved in PBS for 20 min. After washing with PBS, cell images were acquired at ×20 magnification under the microscope equipped with a digital camera or ×100 magnification under the confocal microscope system (for Figure 2). 

#### 4.2.7. Cell Staining with Organelle Staining

HeLa cells (10,000 cells/well in 200 µL medium) were plated into each well of a 96-well glass bottom microplate. After 24 h incubation, the medium in each well was removed and exchanged with 50 µL of sample solution of fluorescent probe **5a** with Lipofectamine 2000 prepared as above, then incubated at 37 °C under the atmosphere of 5% CO_2_ for 2 h. Cells were fixed in 4% paraformaldehyde at room temperature for 20 min. After fixation and washing with PBS, the cell images of fluorescent signal of **5a** were acquired at ×100 magnification under the confocal microscope system (for Figure 3). 

Then, cells were permeabilized with 100 µL of 0.1% Triton X-100 in PBS for 30 seconds. After washing with PBS twice, blocking was carried out with 50 µL of Blocking One-P (Nacalai Tesque Inc., Kyoto, Japan) for 20 min. Organelle stain was performed using concanavalin A (Con A) Alexa Fluor 594 conjugate (Thermo Fisher Scientific Inc., Waltham, MA, USA) for endoplasmic reticulum and HPA from *Helix pomatia* (edible snail) Alexa Fluor 647 conjugate (Thermo Fisher Scientific Inc., Waltham, MA, USA) for Golgi apparatus, respectively. A total of 50 µL of Con A Alexa Fluor 594 conjugate solution at the final concentration of 25 µg/mL in PBS or HPA Alexa Fluor 647 conjugate solution at the final concentration of 7.5 µg/mL in PBS was added to each well and shaken for 1h at the room temperature in the dark, respectively. After washing with PBS, microscopic images at ×100 magnification were obtained under the confocal microscope system (Olympus FV1000). Prior to the organelle staining, fluorescent images stained with fluorescent probes **5a** were obtained, because fluorescence reduced dramatically by treatment of the permeabilization buffer containing Triton X-100 which may dissociate the weak binding between kapakahine probes and the target molecules. The obtained two images stained with **5a** and Con A/HPA could be overlayed manually, because the permeabilization procedure did not affect the cell shapes (Figure S9).

## 5. Conclusions

In this study, we prepared fluorescent probes of kapakahines A and F in a small-scale manner. As mentioned, one of the major difficulties in exploring the mechanisms of action of marine natural products is the limited amounts of available natural compounds for the use of the preparation of chemical probes. In spite of the limited amounts, the fluorescent labeling of kapakahines was successfully carried out and the localization of the fluorescent probes in the cells was clearly visualized. This result indicated that the chemical probes linked to beads for target identification can be prepared on a small scale using the same reaction, as described. 

## Supplementary Materials

The followings are available online at https://www.mdpi.com/1660-3397/19/2/76/s1, Experimental section including Preparation of compounds **4a** and **4b**, Preparation of compounds **5a/b,** Preparation of compounds **6a/b,** Procedure for weighing small amounts of **5a** and **6a**, Preparation of the negative control **4a** for cell staining, Scheme S1: Reaction conditions of the preparation for **4a**, **4b**, **5a/b**, and **6a/b**, Figure S1: UV spectrum of **3a**, **5a** and **6a**, Figures S2 and S3: ESIMS (neg.) of **4a** and **4b**, Figures S4 and S5: HRESIMS (neg.) of **5a/b** and **6a/b**, Figure S6: Microscopic images of HeLa cells (**a**) or P388 cells (**b**) treated with DMSO, **5a/b**, and **6a/b** at 20 µM for 20 h, Figure S7: Microscopic images of HeLa cells (**a**) or P388 cells (**b**) treated with **5a** at 20 µM for 20 h (green, lower lane), Figure S8: Precipitation of Kap A-5-FL (**6a**) on the bottom of the microplate, Figure S9: Reduction of the fluorescent intensity of **5a** in HeLa cells by the permeabilization using 0.1% Triton X-100 in PBS, Figure S10: Overlay images of the organelle stain and the location of **5a** in HeLa cells, Figures S11–S13: HRESIMS (neg.) of **4a**, **5a**, and **6a**, Figures S14–S16: ^1^HNMR spectrum of **4a**, **5a**, and **6a**.
