# Supplementary material for: Small-Scale Preparation of Fluorescently Labeled Chemical Probes from Marine Cyclic Peptides, Kapakahines A and F
Source: Mar Drugs. 2021 Jan 31;19(2):76. doi: 10.3390/md19020076 (PMC7912572; doi:10.3390/md19020076)
Supplement: Supplementary file 1 [file marinedrugs-19-00076-s001.pdf]

Supplementary Information

Small-scale preparation of fluorescently labeled chemical probes from marine cyclic peptides, kapakahines A and F.

Rie Kamihira[a], Yoichi Nakao [a,b]*

[a] *Research Institute for Science and Engineering, Waseda University, 3-4-1 Okubo, Shinjuku-ku, Tokyo 169-8555, Japan*

[b] *School of Advanced Science and Engineering, Waseda University, Shinjuku-ku, Tokyo 169-8555, Japan*

Corresponding author.

E-mail address: ayocha@waseda.jp (Yoichi Nakao) : Tel. & fax: +81-3-5286-3100

Table of contents

Experimental section

Preparation of compounds **4a** and **4b.**

Preparation of compounds **5a/b**.

Preparation of compounds **6a/b**.

Procedure for weighing small amounts of **5a** and **6a**.

Preparation of the negative control **4a** for cell staining.

Scheme S1. Reaction conditions of the preparation for **4a, 4b, 5a/b**, and **6a/b**.

Figure S1. UV spectrum of 5-FL(**3a**), Kap A-5-FL (**5a**) and Kap F-5-FL (**6a**)

Figure S2. ESIMS (neg.) of 5-FL-COOMe (**4a**).

Figure S3. ESIMS (neg.) of 6-FL-COOMe (**4b**).

Figure S4. HRESIMS (neg.) of Kap A-5,6-FL (**5a/b**).

Figure S5. HRESIMS (neg.) of Kap F-5,6-FL (**6a/b**).

Figure S6. Microscopic images of HeLa cells (**a**) or P388 cells (**b**) treated with DMSO, **5a/b**, and **6a/b** at 20 μM for 20h.

Figure S7. Microscopic images of HeLa cells (**a**) or P388 cells (**b**) treated with **5a** at 20 μM for 20h (green, lower lane).

Figure S8. Precipitation of Kap A-5-FL (**6a**) on the bottom of the microplate.

Figure S9. Reduction of the fluorescent intensity of **5a** in HeLa cells by the permeabilization using 0.1% Triton X-100 in PBS.

Figure S10. Overlay images of the organelle stain and the location of **5a** in HeLa cells.

Figure S11. HRESIMS (neg.) of 5-FL-COOMe (**4a**).

Figure S12. HRESIMS (neg.) of Kap A-5-FL (**5a**).

Figure S13. HRESIMS (neg.) of Kap F-5-FL (**6a**).

Figure S14. ^{1}HNMR spectrum (600 MHz, methanol-d_4) of 5-FL-COOMe (**4a**).

Figure S15. ^{1}HNMR spectrum (600 MHz, methanol-d_4) of Kap A-5-FL (**5a**).

Figure S16. ^{1}HNMR spectrum (600 MHz, methanol-d_4) of Kap F-5-FL (**6a**)

Preparation of compounds 4a and 4b.

To the MeOH solution of 6-{fluorescein-5 (or 6)-carboxamido}hexanoic acid, succinimidyl ester (5 or 6-SFX: **3a/b**, 2.9 mg, 4.94 μmol in 1.48 mL of MeOH, Thermo Scientific) was mixed with 1.52 mL of triethylamine (TEA, 10.9 mmol). The reaction mixture was stirred at room temperature in the dark for 19h. The reaction was monitored by TLC (Thin layer chromatography). The reaction was quenched with excess amounts of MeOH and dried. The reaction mixture was subjected to reversed-phase HPLC [COSMOSIL 5C18-AR II (φ10×250 mm), flow rate; 2 mL/mL, isocratic elution with 40% acetonitrile 0.05% TFA, detection; 220 nm, TOSOH UV8011] to yield two fractions containing 5-FL-COOMe (**4a**, 0.7 mg) and 6-FL-COOMe (**4b**, 0.8 mg), respectively. Compounds **4a** and **4b** were regioisomers with the branch in the fluorescein at C-5 and C-6 respectively. Both compounds gave the same $[M-H]^-$ ion peaks at *m/z* 502 corresponding to $C_{28}H_{24}NO_8$ in the ESIMS respectively.

Preparation of compounds 5a/b.

A portion (200 μL) of **3a/b** in *N,N*-dimethylformamide (DMF) (20 mg/mL, 6.8 μmol, Thermo Scientific) was added dropwise to a kapakahine A (**1**, 1.0 mg, 0.95 μmol) dissolved in 50 μL of DMF. The reaction mixture was stirred at room temperature in the dark for 21h. After quenching with MeOH, the reaction mixture was subjected to ODS flash column chromatography eluting with 20, 50, 70, and 100% MeOH to yield 6 fractions. The 100% MeOH eluting fraction was further purified by reversed-phase HPLC [COSMOSIL 5C18-AR II (φ10×250 mm), flow rate; 2 mL/min, isocratic elution with 55% acetonitrile, detection; 220 nm , TOSOH UV8011] to yield Kap A-5,6-FL (**5a/b**: 340 μg) as a mixture of two regioisomers. In the ESIMS analysis, **5a/b** gave an $[M-H]^-$ ion peak at *m/z* 1522.6731 (calcd. $C_{85}H_{92}N_{11}O_{16}$ 1522.6729).

Preparation of compounds 6a/b.

Kapakahine F (**2**) (0.5 mg, 0.71 μmol) in 33.3 μL DMF was added to 66.6 μL of reagent **3a/b** in DMF (20 mg/mL) and was stirred at room temperature in the dark for 20h. After quenching with MeOH, the reaction mixture was subjected to ODS flash column chromatography eluting with H_2O, 20, 50, 70, and 100% MeOH to yield 5 fractions. The fraction eluting with 100% MeOH was further purified by reversed-phase HPLC [COSMOSIL 5C18-AR II (φ10×250 mm), flow rate; 2 mL/min, isocratic eluting with 50% acetonitrile, detection; JASCO FP-2020 Plus intelligent fluorescence detector, excitation 502 nm, emission 527nm] yielding Kap F-5,6-FL (**6a/b**, 79μg). Kap F-5,6-FL (**6a/b**) gave an $[M-H]^-$ ion peak at *m/z* 1171.4580 (calcd. $C_{67}H_{63}N_8O_{12}$ 1171.4571).

Procedure for weighing small amounts of 5a and 6a

The accurate weights of Kap FLs were obtained using microbalance as follows. As the standard, we prepared a solution (2 mg/mL) of cyclosporin A in MeOH, a commercially available cyclic peptide. Each portion (1, 5, 25, 50 μL) of the solution were added to the weighed dry tin dish, respectively. These dishes were transferred into a desiccator, decompressed with a vacuum pump. After drying, each dish was weighed. The weight of the compound was obtained by subtraction of the weight of the dried tin dish from that with the compounds. When a calibration curve was drawn based on the calculated weighs, a good determination constant (R^2) and a straight line were obtained. The kapakahine probes were weighed following the procedure above. First, these probes were dissolved in a fixed amount of MeOH, and 2 or 5 μL of the solution was put on the weighed tin dish. The weights of the kapakahine probes were obtained by subtraction.

Preparation of the negative control 4a for cell staining.

Initially, the fluorescent dye **3a/b** itself was used as the negative control in the preliminary cell staining experiments. However, the cells were strongly stained with **3a/3b**, presumably because of the NHS group of **3a/b** easily reacted with the ε-amino group of Lys residue of proteins existing on the surface or inside the cells. Therefore, fluorescent methyl ester **4a** was used as the negative control for the cell staining experiments.

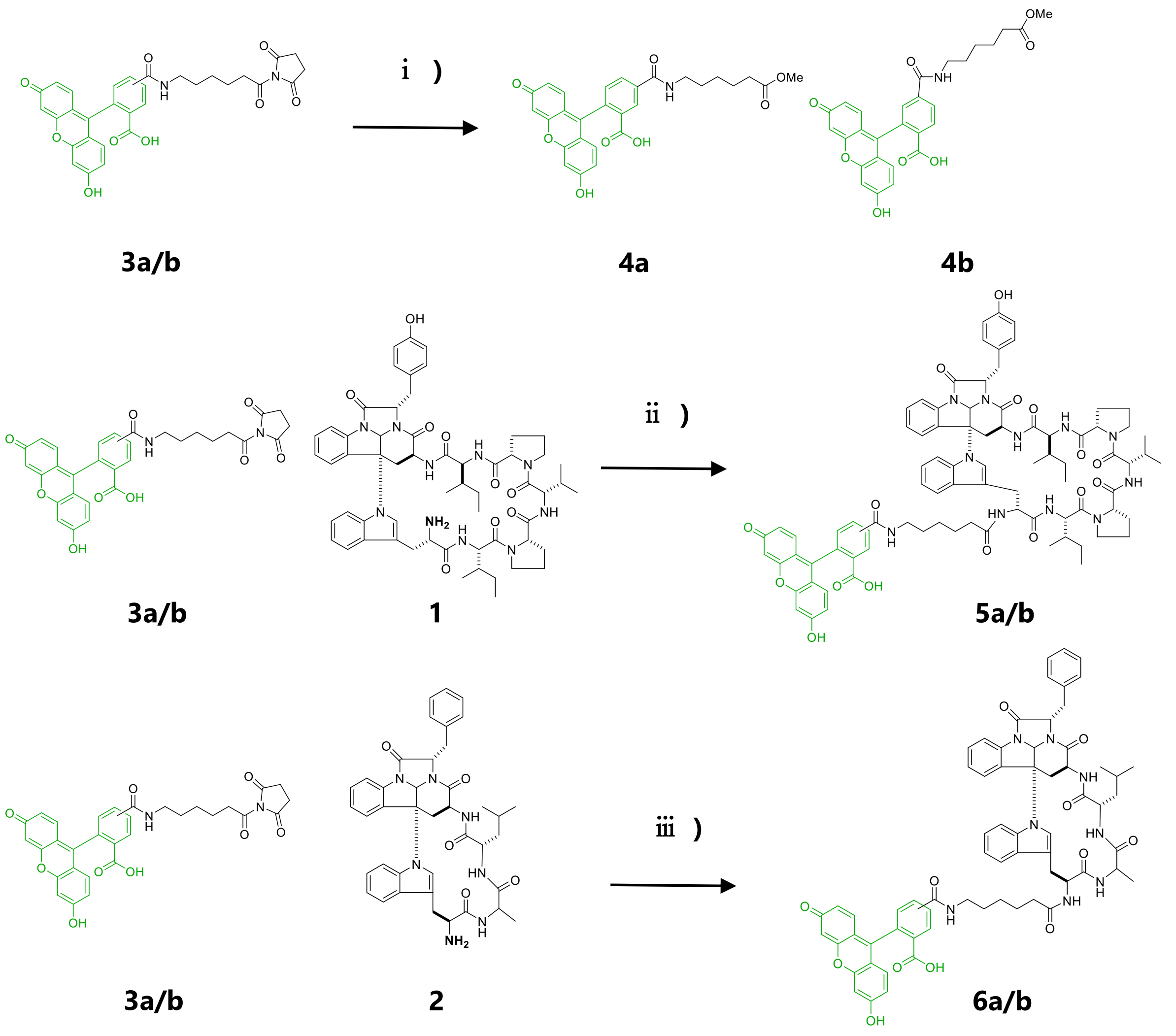

Scheme S1. Reaction conditions of the preparation for **4a**, **4b**, **5a/b,** and **6a/b**. (i) MeOH, triethylamine rt, 19h ; (ii) DMF, rt, 20h ; (iii) DMF, rt, 21 h.

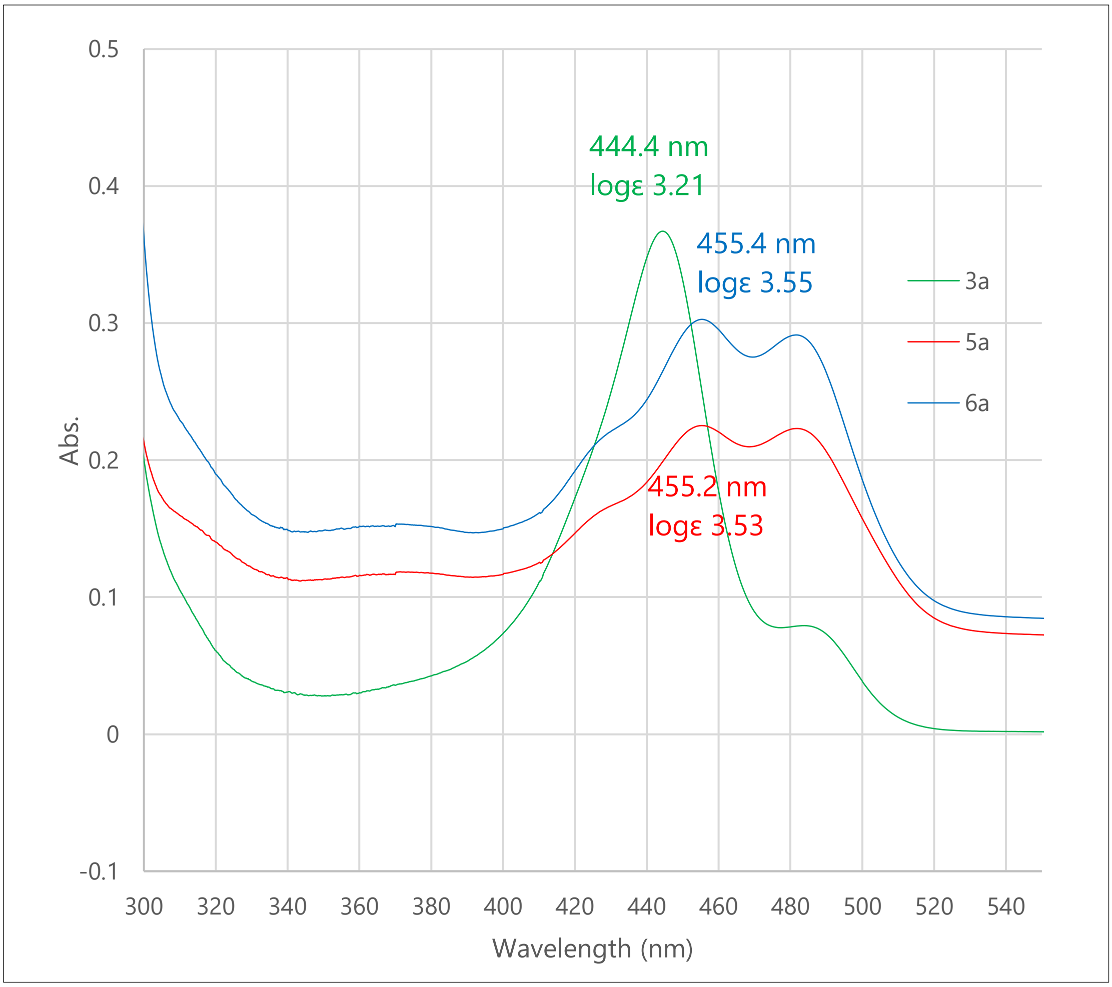

Figure S1. UV spectrum of 5-FL(**3a**), Kap A-5-FL (**5a**) and Kap F-5-FL (**6a**).

.

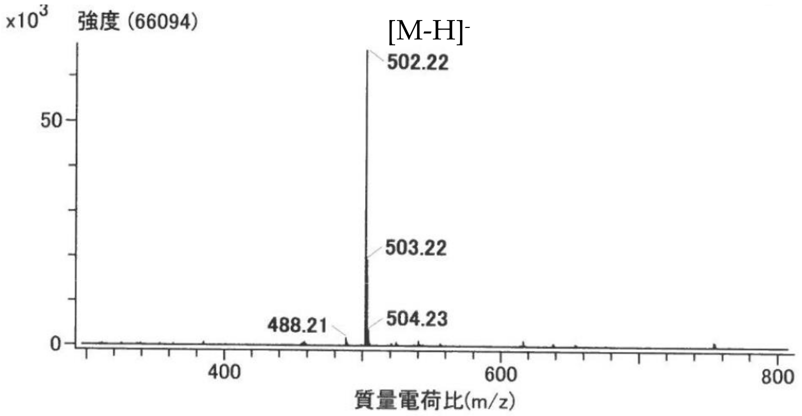

Figure S2. ESIMS (neg.) of 5-FL-COOMe (**4a**).

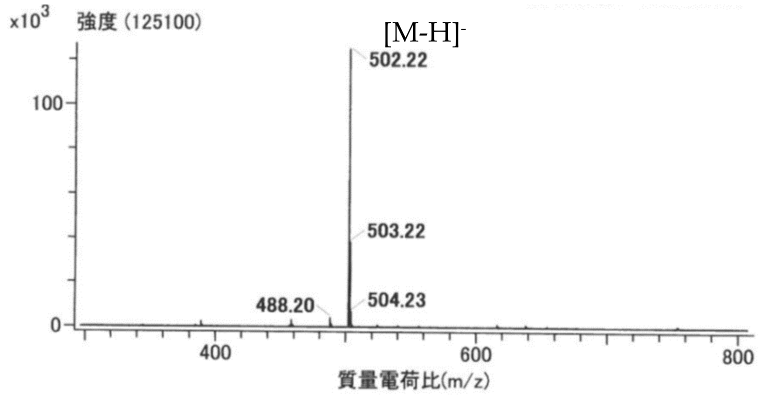

Figure S3. ESIMS (neg.) of 6-FL-COOMe (**4b**).

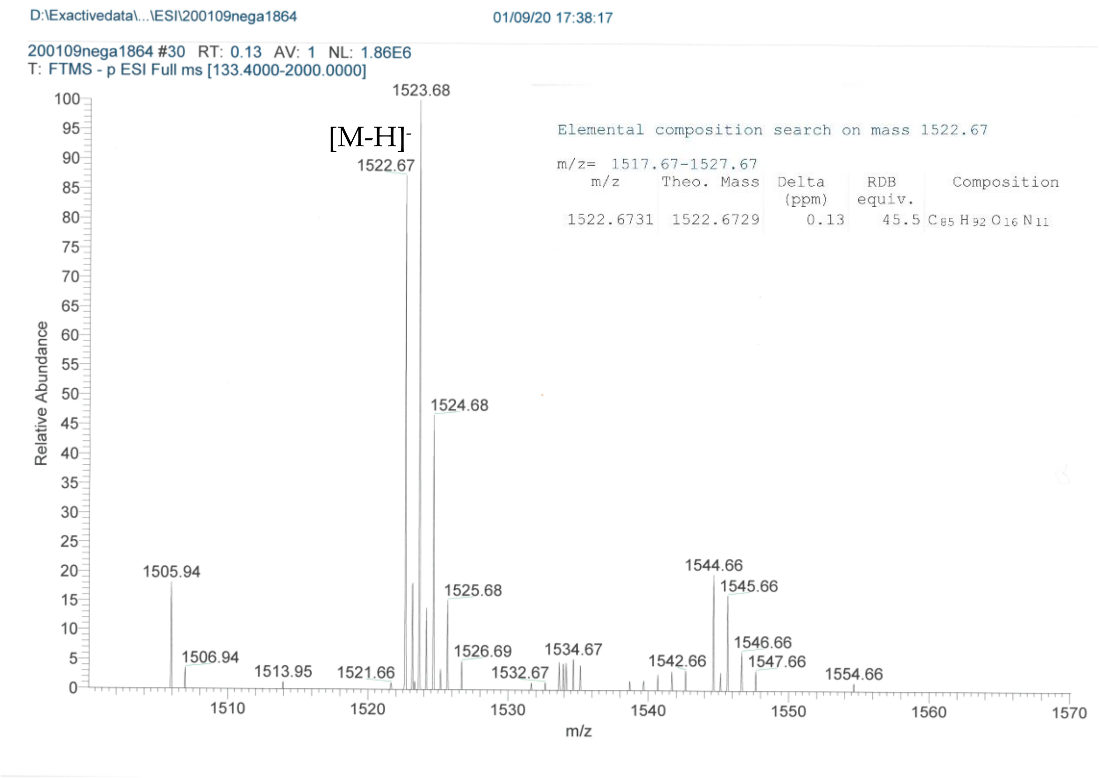

Figure S4. HRESIMS (neg.) of Kap A-5,6-FL (**5a/b**).

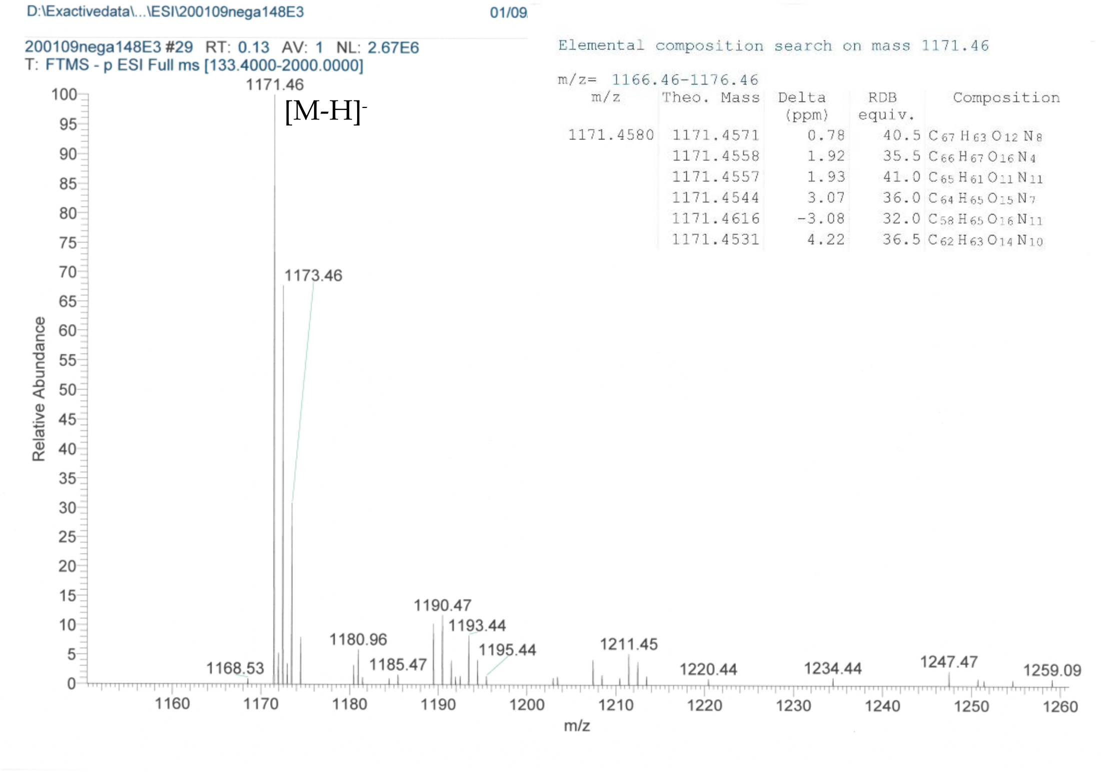

Figure S5. HRESIMS (neg.) of Kap F-5,6-FL (**6a/b**).

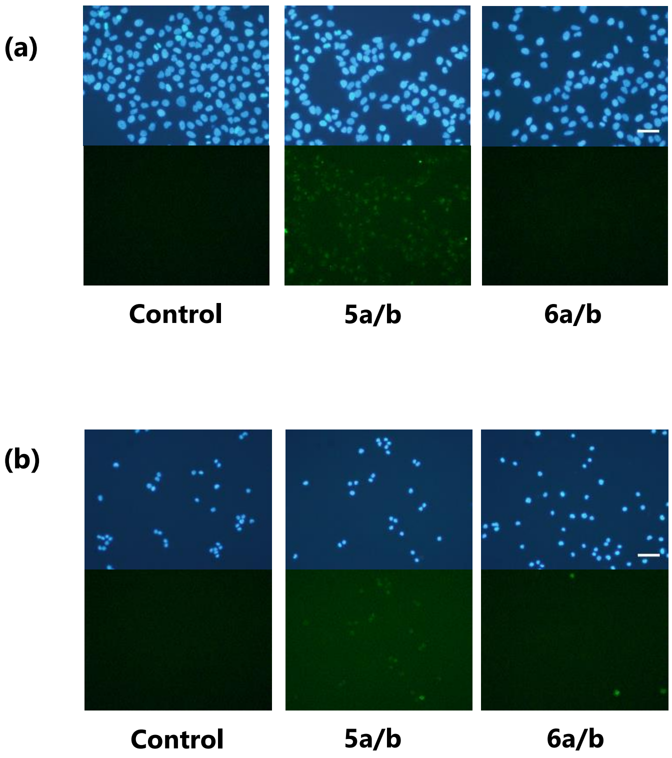

Figure S6. Microscopic images of HeLa cells (**a**) or P388 cells (**b**) treated with DMSO, **5a/b** and **6a/b** at 20 μM(green) for 20h. Nuclei stained with Hoechst 33342 (blue), magnification ×20, scale bar: 50 μm. The brightness and contrast were equally adjusted for all the green fluorescent images.

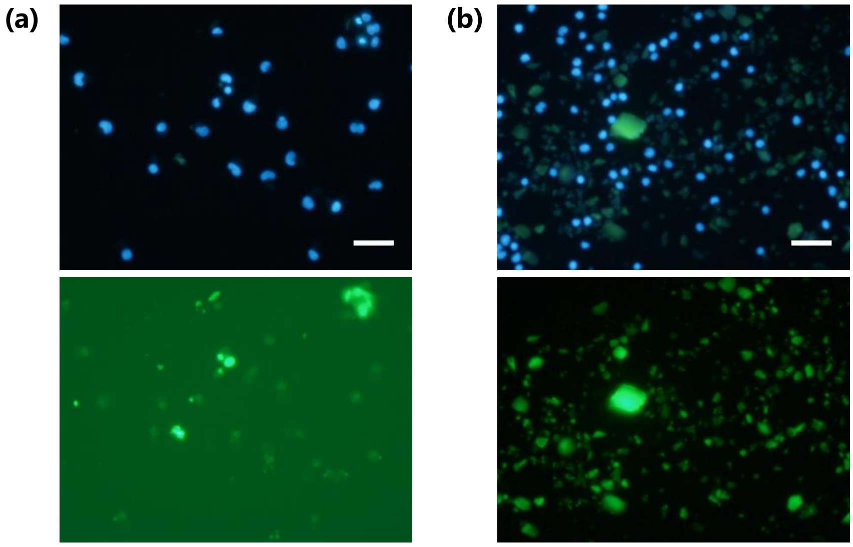

Figure S7. Microscopic images of HeLa cells (**a**) or P388 cells (**b**) treated with **5a** without Lipofectamine 2000 at the concentration of 20 μM for 20h (green, lower lane). Nuclei stained with Hoechst 33342 (blue, upper lane), magnification ×20, scale bar: 50 μm. The brightness and contrast were equally adjusted for all the green fluorescent images.

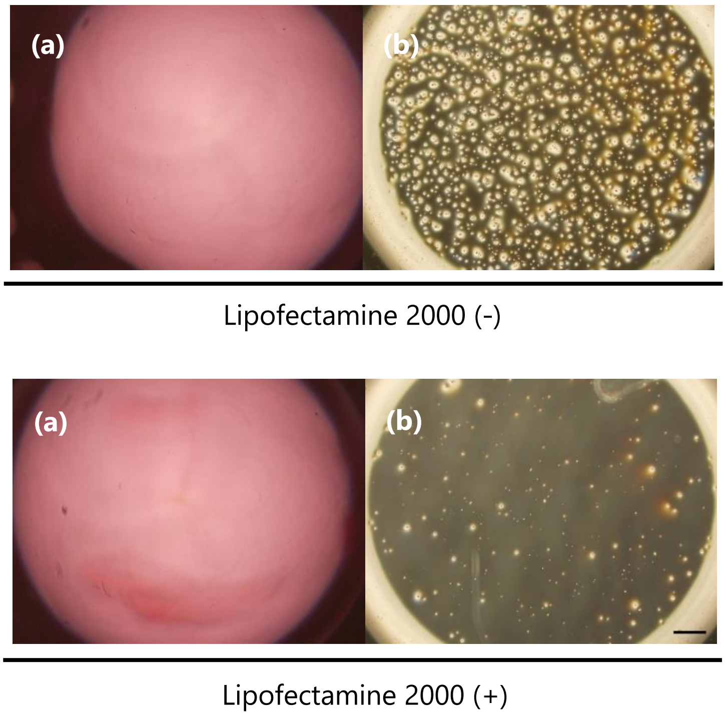

Figure S8. Precipitation of Kap A-5-FL (**6a**) on the bottom of the microplate. Lipofectamine 2000 (-) (upper) and Lipofectamine 2000 (+) (lower). The supernatant of the medium mixture containing Kap A-5-FL (**5a**) and Lipofectamine 2000 (**a**) the precipitation of the Kap A-5-FL (**5a**) on the glass bottom of the microplate after removal of the cultural medium (**b**), magnification ×4, scale bar: 200 μm.

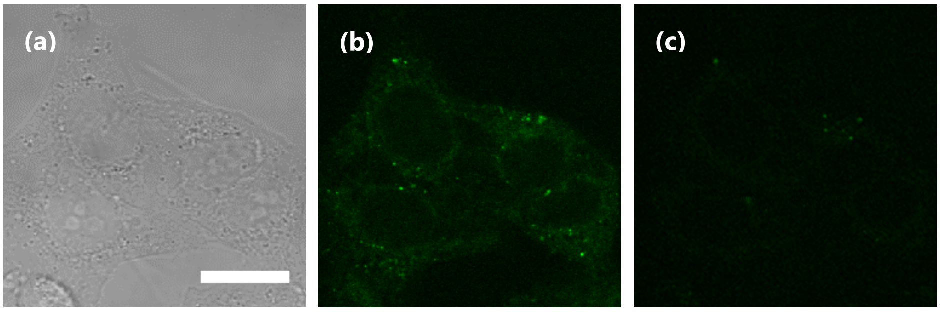

Figure S9. Reduction of the fluorescent intensity of **5a** in HeLa cells by the permeabilization using 0.1% Triton X-100 in PBS. After fixation in 4% PFA bright field (**a**), green fluorescence (**b**), green fluorescence after permeabilization using 0.1% Triton X-100 in PBS (**c**), magnification ×100, scale bar: 20 μm. The brightness and contrast were equally adjusted for all the green fluorescent images.

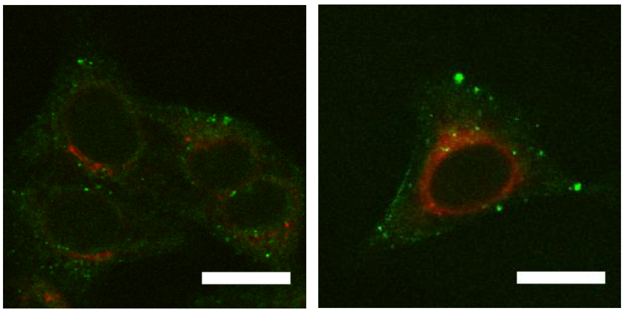

Figure S10. Overlay images of the organelle stain and the location of **5a** in HeLa cells. Confocal microscopic images of HeLa cells incubated with **5a** in Lipofectamine 2000 for 2h (green). Since green fluorescence intensity decreases during organelle staining, organelle staining was performed after taking photograph of the green fluorescence of fixed cells using a confocal microscope. Organelle staining was performed using Lectin HPA (red, left) for Golgi apparatus and concanavalin A (red, right) for endoplasmic reticulum. The brightness and contrast were adjusted equally for all the green fluorescent images.

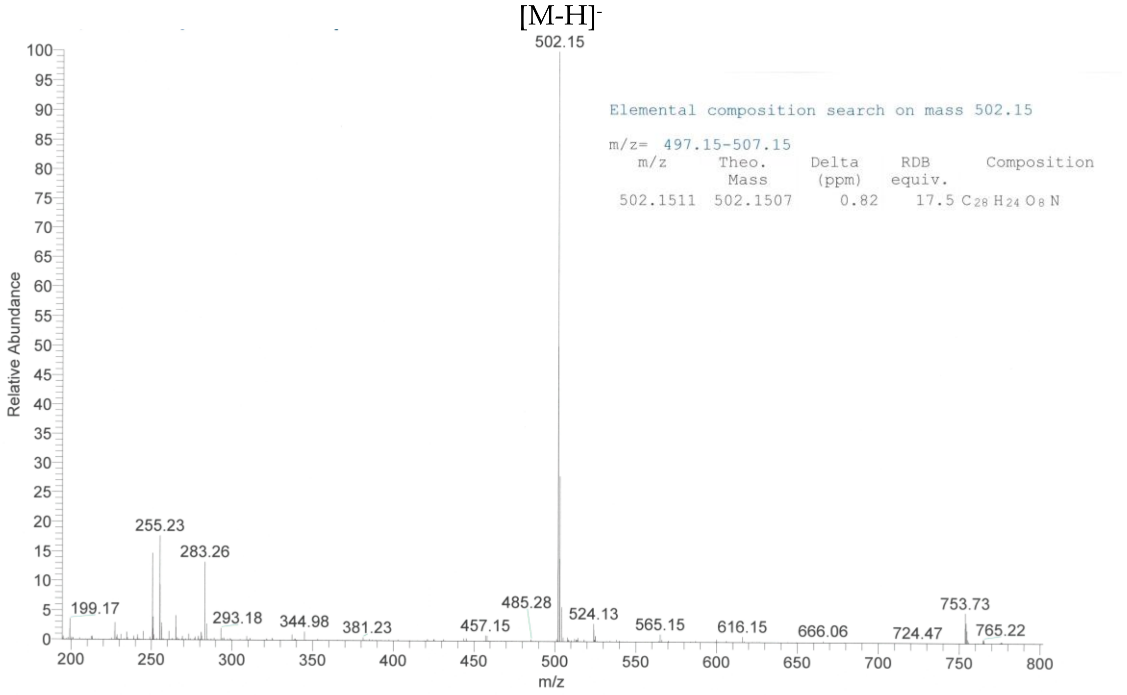

Figure S11. ESIMS (neg.) of 5-FL-COOMe (**4a**).

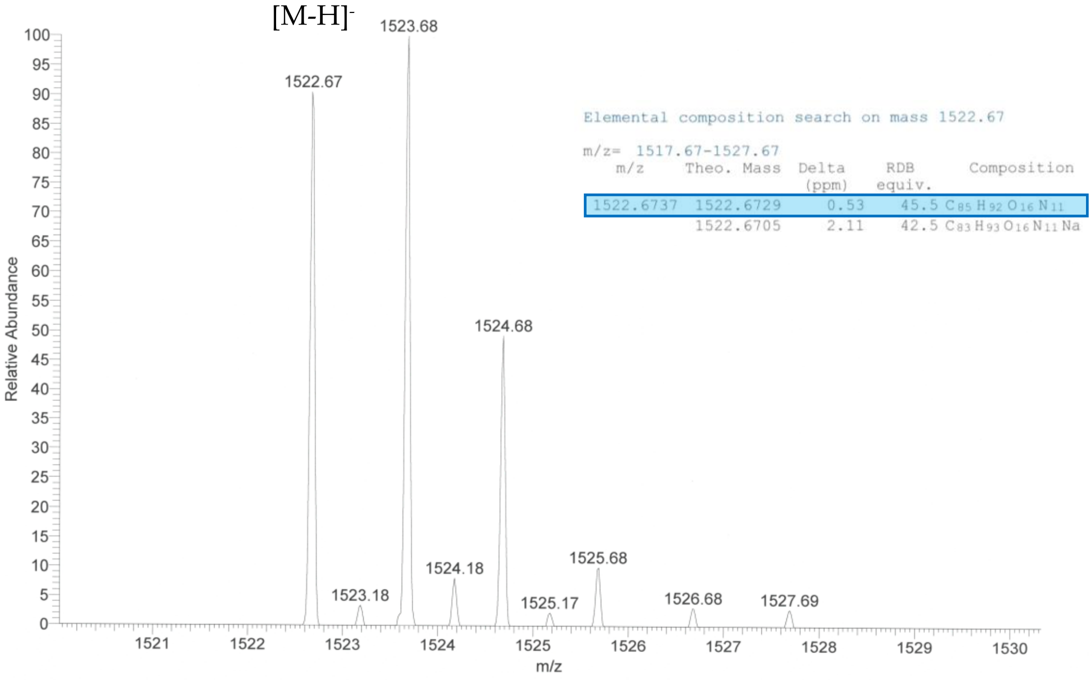

Figure S12. HRESIMS (neg.) of KapA-5-FL (**5a**).

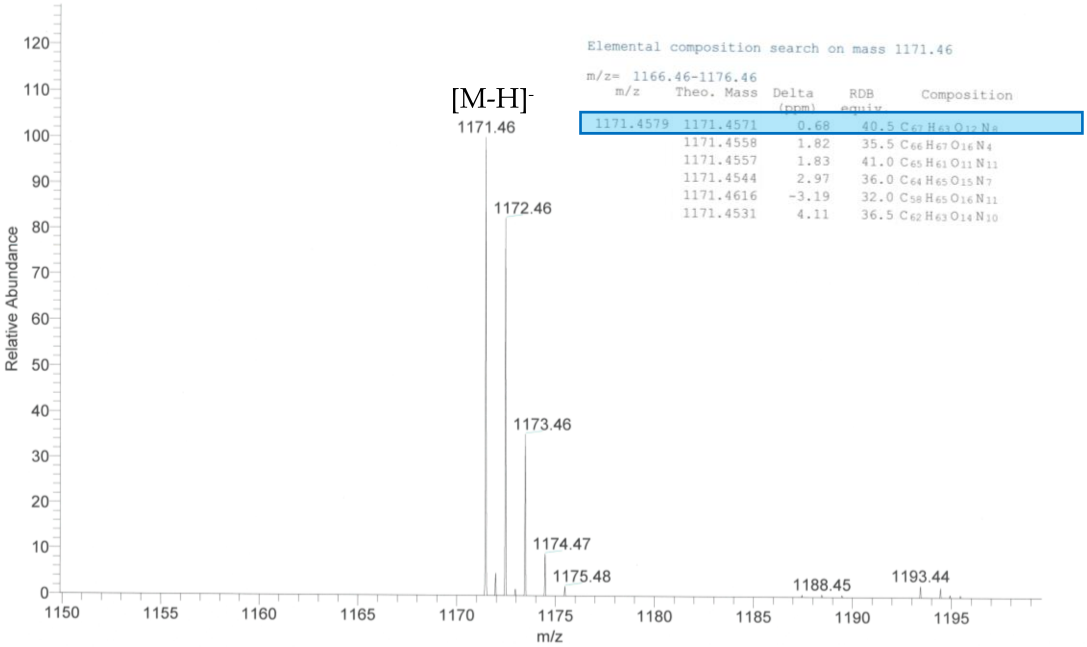

Figure S13. HRESIMS (neg.) of Kap F-5-FL (**6a**).

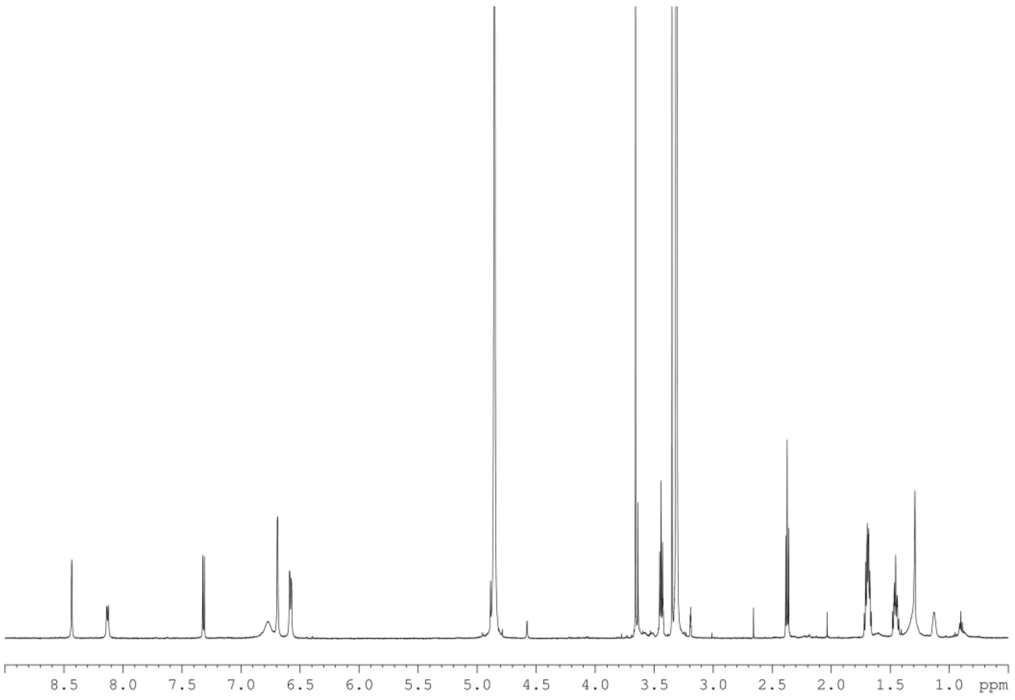

Figure S14. ^{1}HNMR spectrum (600 MHz, methanol-*d4*) of 5-FL-COOMe (**4a**).

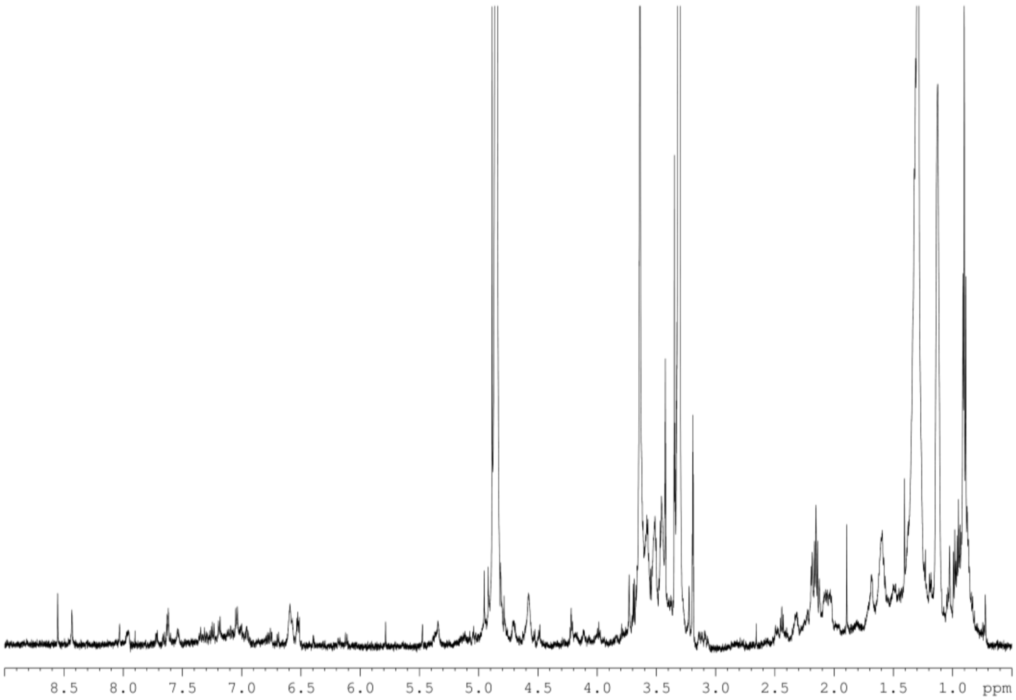

Figure S15. ^{1}HNMR spectrum (600 MHz, methanol-*d4*) of Kap A-5-FL (**5a**).

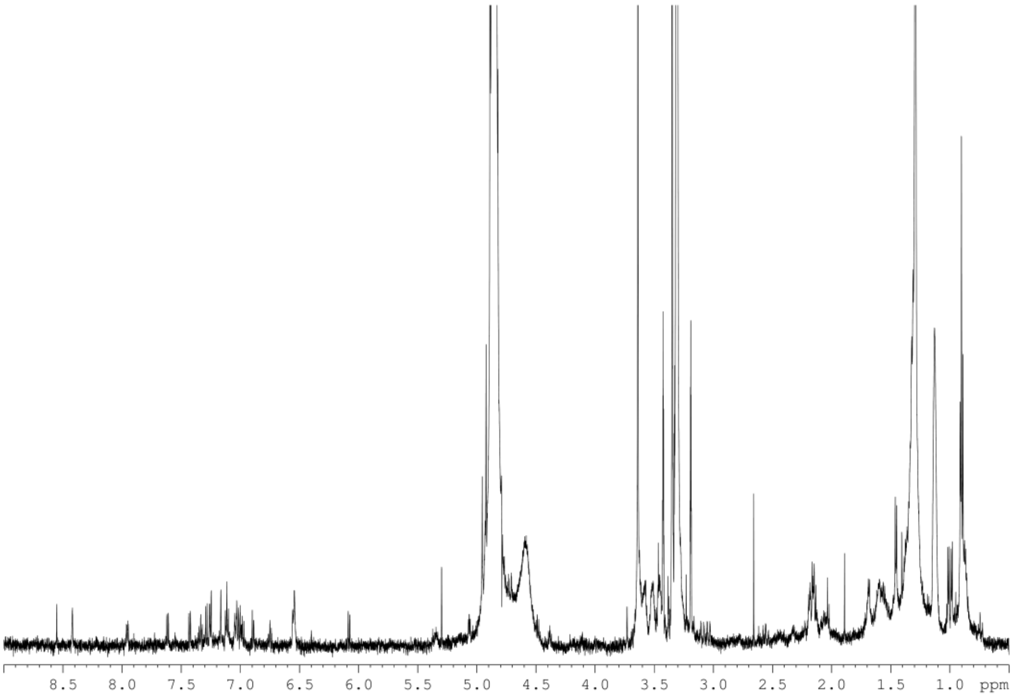

Figure S16. ^{1}HNMR spectrum (600 MHz, methanol-*d*$_4$) of Kap F-5-FL (**6a**).
